# Poor Dissociation of Patient-Evaluated Apathy and Depressive Symptoms

**DOI:** 10.1155/2012/846075

**Published:** 2012-05-30

**Authors:** Progress Njomboro, Shoumitro Deb

**Affiliations:** ^1^Department of Psychology, University of Cape Town, Private Bag Rondebosch, Cape Town 7701, South Africa; ^2^Department of Psychiatry, Division of Neuroscience, University of Birmingham, Birmingham B15 2TT, UK

## Abstract

Apathy has traditionally been conceptualised as part of depression. The appropriateness of this conceptualisation has now been questioned, with the realization that apathy constitutes a distinct neuropsychiatric condition, with separate rehabilitation and patient-care implications to depression. Research on the relationship between apathy and depression has, however, produced mixed results. One reason for this inconsistency may lie behind who does the apathy evaluation. In this study we investigated whether the relationship between apathy and depression would differ when apathy was evaluated by the patients or an informant. A total of 49 brain damaged patients were assessed on self- and informant-rated Apathy Evaluation Scales. The relationship between the apathy scores and depressive symptoms was then investigated. Patient-rated, and not informant-rated apathy significantly correlated with depression. We discuss the implication of these results on the relationship between the two neuropsychiatric conditions and also in relation to the utility of patient self-evaluations in apathy.

## 1. Introduction

The position of apathy as a distinct syndrome in both clinical practice and research is still uncertain and less clearly defined. Generally, apathy is conceptualised as constituting a significant loss of motivation [1]. For diagnostic purposes, this loss of motivation must be present for at least four weeks and should manifest in at least two of three dimensions of apathy involving reduced overt acts, cognitive activity, and affective responses related to goal directed behaviour [2]. The clinical importance of apathy is demonstrated through its association with reduced patient independence, social integration, rehabilitation success, and increased caregiver burden [3] and its high prevalence in patients suffering neurological change [4]. For instance, incidence of between 17–70% has been reported in Parkinson's disease [5, 6], and incidence of between 46 and 71% has been reported in patients with traumatic brain injury [7, 8]. Similar high incidence rates have been reported in Alzheimer's disease, frontotemporal dementia, progressive supranuclear palsy, and stroke [9–11]. See also [12] for a review.

Much of the debate on apathy in the past decade has focused on its nosological position, particularly its relation to depressive symptoms [6, 13–16]. Traditionally, apathy has been viewed as a symptom of depression. Clinically, the two disorders are related in that they significantly overlap on symptom dimensions related to loss of interest, anhedonia, and reduced activity [9, 17]. This debate on the relationship between apathy and depression still remains important. For instance, the inclusion of a dimension of “diminished interest” as a core symptom of depressive disorder is now queried, with some suggesting that this symptom dimension relates more to apathy than depression [5]. Also worryingly, one study found that, in 33% of Parkinson's disease patients suffering from minor depression and 8% suffering from major depression, this diagnosis was made solely on the basis of loss of interest in the absence of depressed mood [18]. Evidence also suggests that quite often apathetic patients are misdiagnosed as depressed by practitioners, and consequently wrongly prescribed antidepressants [19].

Some of the distinctions between apathy and depression are now widely appreciated. For example, while depression is a mood disorder involving emotional pain, negative cognitive biases, despair, hopelessness, and pessimism, apathy is primarily a disorder of motivation, marked by general lack of interest, concern, or care about almost anything [9, 20]. Furthermore, apathy and not depression is usually associated with cognitive impairments, which indicate separate neurocognitive profiles for the two conditions [16, 21–24]. There is also evidence that separate brain circuits are involved in apathy and depression [25, 26], further strengthening the view that the two are different neuropsychiatric disorders. Despite these noted differences, some studies have reported significant symptom overlap between the two syndromes [15] or shown a significant association between depression and apathy symptoms [24]. In one study on Parkinson's patients [27], apathy coexisted with depression in 43% of the patients. Other studies have found a modest or low correlation between the two disorders [16, 28]. For alternative results, see [29].

A number of possible explanations, ranging from the use of different assessment tools and clinical samples across studies; frequency of both apathy and depression in patients with neurological damage (e.g., [27, 30, 31]); the use of different cutoffs on apathy scales (see [10, 32, 33]), may account for the variations in the reported relationships between apathy and depression. Some depression scales also treat apathy as a symptom of depression [28, 34]. For example, the HAMD-21 has an item on “Work and activities” that specifically target apathy [35]. Moreover, the concepts of apathy and depression both share the predicate of “reduced volition,” which implies some phenomenological overlap between the two [9, 19, 32].

The use of patient or informant rated apathy scores across different studies may also account for the mixed findings on the relationship between apathy and depression. Evidence suggests that brain-damaged patients are poor at self-evaluating their apathy symptoms [36]. So far, no study has investigated how using patient or informant-rated apathy scores mediates the relationship that is found between apathy and depression. This factor is crucial in light of the overlap between apathy symptoms, cognitive deficits, and lack of insight commonly seen in patients suffering neurological change [5, 6, 37]. It is possible differential apathy ratings between patients and informants account for part of the inconsistent research results in this area.

This study focuses on the relationship between apathy and depression in patients with acquired brain damage. Specifically, we investigated whether this relationship is mediated by the use of patient or informant-rated apathy scores. The self (AES-S) and informant-rated (AES-I) versions of the Apathy Evaluation Scale (AES; [10]) and the apathy section of the Neuropsychiatric Inventory (NPI; [38]) were used to assess apathy. Correlations with an apathy score on the NPI were used to enable the independent examination of the relationships between the two AES versions. The NPI and AES are the most widely used and psychometrically robust scales for assessing apathy (see review by [39]). We hypothesised that patient and informant-rated apathy would relate differently to depression. The level of depressive symptoms was evaluated using the Hospital Anxiety and Depression Scale (HADS-D; [40]) and the Depression Inventory (BDI; [41]). Most studies investigating the relationship between apathy and depression have tended to use homogenous patient samples (e.g., [5, 6, 32]). The weakness in such studies is that the relationship between apathy and depression may interact in some specific ways with the aetiological process. For that reason, our aim was to take brain-damaged patients from a wide range of aetiologies, in order to give a broad sample of possible relations between depression and apathy. We also obtained an executive function measure on the Tower of Hanoi task (ToH), to assess patients' cognitive control.

## 2. Methods

### 2.1. Participants

#### 2.1.1. Patients

A total of 49 brain-damaged patients at least 6 months after-injury took part in the study (see Table 1 for patient characteristics). Patients were recruited from clinics and rehabilitation centres in the West Midlands, United Kingdom. Informed written consent was obtained from all participants. Lesion data was available on 46 patients. In 25 patients, the lesions were detected using voxel-based morphological analysis in SPM5 (http://www.fil.ion.ucl.ac.uk/spm/software/spm5). The images were first segmented into grey matter, white matter, and cerebrospinal fluid (CSF), and the resulting tissue classes images were normalized without modulation (i.e., to compensate for the effect of spatial normalization). Images were smoothed with a Gaussian kernel of 2 × 2 × 2 mm. Significant changes were based on one sample *t*-tests with 3 covariates: healthy grey/white matter versus patient grey/white matter, age, and gender. All areas were FWE corrected with *P* = 0.05 and an extent threshold specifying that only significant blobs containing ≥100 voxels be included in the lesion. In the other 21 patients, data on lesion location was obtained from reports in patient files on scans performed before admission to rehabilitation units. Twenty-two patients had bilateral frontotemporal lesions, 7 had right fronto-temporal lesions, 7 had left frontotemporal lesions 1 had bilateral parietal lesions, 4 had right parietal lesions, and 5 had left parietal lesions.

#### 2.1.2. Informants

Patients' caregivers gave evaluations on the AES-I and the NPI. These informants were either relatives, or care-workers (in the case of patients in rehabilitation units) that interacted with the patient on a daily basis, and in all the cases, had known the patients for at least 5 months. On both instruments, the principal investigator sat with the informants and read out each of the items, seeking clarification on responses that were not clear. The Birmingham and Solihull Research Ethics Committee approved the research procedures for this project, and all participants gave informed written consent.

### 2.2. Apathy Evaluation

Patients were evaluated for levels of apathy using the self (AES-S) and informant- (AES-I) rated versions of the Apathy Evaluation Scale [10] and also on the apathy section of the Neuropsychiatry Inventory (NPI [38]).

#### 2.2.1. The Apathy Evaluation Scale [10]

The AES is an 18-item scale that assesses behavioural, emotional, and cognitive aspects of apathy. Each item (e.g.,* s/he gets things done during the day/I get things done during the day)* is rated on a scale of 1 *(Not at all)* to 4 *(A lot)*. A higher AES score indicates more apathy. The scale has been widely used for research and shown to demonstrate good psychometric properties [10, 42, 43]. We used an AES cut-off score of 38 (AES-S) and 40 (AES-I) to determine whether patients were apathetic on not [10].

#### 2.2.2. The Neuropsychiatric Inventory [38]

The neuropsychiatric inventory (NPI) evaluates 10 specific behavioural domains; delusions, hallucinations, agitation, depression, anxiety, elation, apathy, disinhibition, irritability, and aberrant motor behaviour and gives a subscore for each of these domains. If a specific neuropsychiatric symptom is present, it is rated on a 4-point frequency scale (occasionally = 1; often = 2; frequently = 3; very Frequently =  4) and a separate 3-point severity scale (mild = 1, moderate = 2, marked = 3). Multiplying the frequency rating score by the severity rating score produces the subscale score for each behavioural domain. For purposes of this study, only the apathy subscale was used. It includes items such as showing loss of interest, lacking motivation, less spontaneous, less affectionate, less enthusiastic, lacking in emotions, and not caring about doing new things. The subscale has demonstrated good internal consistency (*α* = .87-.88), test-retest (*r* = .74 for frequency), and interrater reliabilities (*r* = .89 for severity and *r* = .98 for frequency [38]. The subscale has also been used to assess apathy in a number of studies (e.g., [33, 44]). We took NPI scores above 4 as indicative of the presence of apathy [45].

### 2.3. Depressive Symptoms Evaluation

The presence and level of depressive symptoms were evaluated using [40]'s Hospital Anxiety and Depression Scale (HADS-D) and the Beck Depression Inventory (BDI).

#### 2.3.1. Beck Depression Inventory [41]

The BDI has enjoyed wide usage as a valid and reliable assessment tool for depressive symptoms [46]. Patients evaluate themselves by putting a circle on one of the 4 likert-type statements making each of the 21 items making up the inventory. Scores on each statement range from 0 (absence of that aspect of depression) to 3 (most severe). For example, *“I do not feel sad*” carries a score of 0, *“I feel sad”* a score of 1, *“l am sad most of the time and l cannot snap out of it”* a score of 2, and “*I am so sad or unhappy that l cannot stand it,”* a score of 3. The BDI score is obtained by summing up all the scores. A higher score suggests increased symptoms severity. Cut-off scores on the BDI are not consistent across studies [47], but in this study we classified scores at or above 11 as indicative of the presence of depression [48].

#### 2.3.2. Hospital Anxiety and Depression (Zigmond and Snaith, 1983)

The HADS-D is a 14-item self-screening paper and pen questionnaire that assesses levels of anxiety and depressive symptoms. It was initially designed for hospital general medical outpatients but is now extensively used in primary care and research. 7 items on the test are relevant to depressive symptoms, and the other 7 to generalised anxiety. Each item has 4 possible responses scored on a scale that ranges from 0 to 3. For example, the item *“I still enjoy the things I used to enjoy,”* has 4 possible responses: *“definitely as much (0), Not quite so much (1), Only a little (2), Hardly at all (3).” *This gives a possible maximum score of 21 for either depression or anxiety. For purposes of this study we only used the depression section of the scale and adopted a cut-off score of 9 to distinguish between depressed and non-depressed patients [40].

### 2.4. Executive Function Measure

#### 2.4.1. The Tower of Hanoi Task

**Figure 1:**
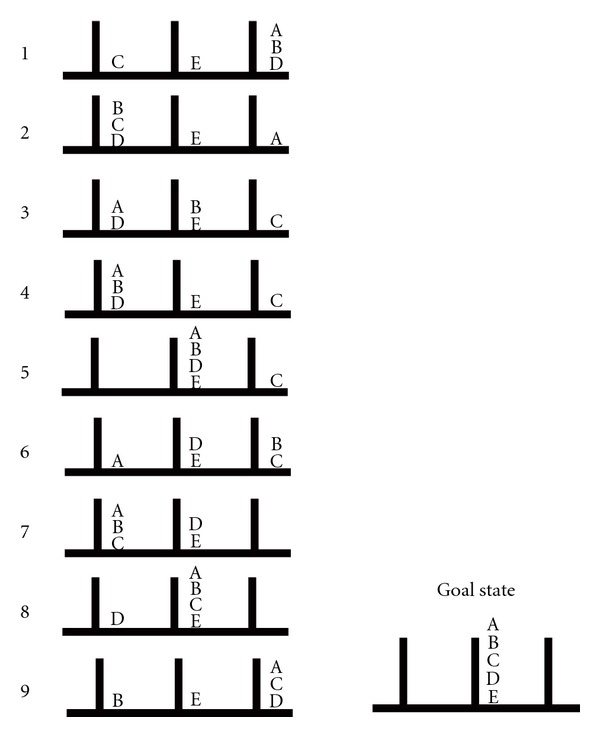
(*Adopted from Goel and Grafman, 1995*). Start states for 9 of the 10 ToH trials used in the study. The tenth trial (not included in the diagram) had a start state in which discs A to E were all placed on the right peg, and it was administered after the 9th trial.

The ToH puzzle provided a cognitive measure of executive function. Solving the puzzle requires the use of forward planning and capacities related to anticipatory, insightful means end problem-solving strategies. A series of 10 ToH trials of varying levels of difficulty were administered to each of the participants. The puzzle consisted of a flat wooden board with three vertical pegs of equal height and diameter fixed equidistantly from each other and five wooden disks (disks A, B, C, D, and E). On each of the 10 trials, the disks were placed in some predetermined arrangement on the pegs (start state), and participants had to rearrange the disks until they were all staked in a descending order according to size on the middle peg (the goal state; see Figure 1 below). Participant had to follow three rules: (a) only one disk could be moved at a time, (b) any disk not being moved had to remain on a peg, and (c) a larger disk could not be placed on top of a smaller disk. The number of trials successfully completed on the task provided the ToH score. For various reasons, scores on the ToH were obtained for 25 patients.

## 3. Results

Data on all the apathy and depression scales was available for 40 patients. Data for patients who did not have scores on all the scales was excluded from analysis. Where data failed to meet the assumptions for the use of parametric tests, nonparametric test equivalents were performed.

### 3.1. Apathy

**Figure 2:**
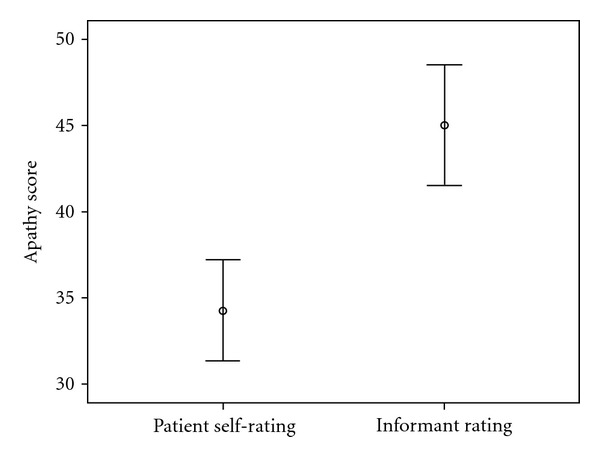
Distribution of self- and informant-rated levels of apathy.

Patient self ratings on the AES-S ranged from 20 to 57 (mean = 34.27, SD = 9.29). Informant ratings on the AES-I ranged from 23 to 69 (Mean = 45.05, SD = 11.06).  Figure 2 shows the distribution of scores on the AES-S and AES-I.

Based on the patients' self evaluations on the AES-S, only 6 (15%) met criteria for the presence of apathy, and 34 (85%) patients self-evaluated themselves as not apathetic. On the other hand informants rated 23 (57.5%) of the patients as apathetic on the AES-I and 17 (42.5%) patients as not having apathy. Wilcoxon signed-rank tests performed on the data to compare AES-S and AES-I scores showed that patients evaluated their apathy levels significantly lower (Mdn = 33.5) than their informants (Mdn = 44.0), *T* = 18.8, *P* < 0.001, *r* = − .47. The effect size (*r* = − .47) is large enough to suggest that this finding is reliable.

Informant ratings on the NPI apathy subscale evaluated 24 (60%) patients as having significant apathy symptoms, and 16 (40%) as not apathetic. Informant-rated AES-I and NPI apathy scores were positively correlated (*rs* = .71, *P* < 0.001). There were no significant correlations between patient-rated apathy scores on the AES-S and informant-rated apathy scores on both the AES-I (*rs* = .27, ns) and the NPI (*rs* = .26, ns; *see* Table 2).

### 3.2. Depression

Two (5%) patients met criteria for the presence of depression on both the BDI and the HADS-D and 38 (95%) had no significant depressive symptoms. There was a significant positive correlation between depressive symptoms scores on the HADS-D and the BDI (*rs* = .55, *P* < 0.001).

### 3.3. Apathy and Depression

Patients' self-rated (AES-S) apathy scores positively correlated with depressive symptoms scores on the HADS-D (*rs* = .48, *P* < 0.01) and the BDI (*rs* = .42, *P* < .01).

There were no significant correlations between informant-rated apathy scores on the AES-I and NPI and depressive symptoms scores on the HADS-D and BDI (see Table 2).

### 3.4. Executive Function

Spearman's rho tests showed a significant negative correlation between ToH scores and AES-I apathy scores (*rs* = −.523, *P* < 0.01). These results showed that the higher the level of apathy in patients, the more they were impaired on the task.

## 4. Discussion

Patient-rated apathy positively correlated with depressive symptoms. No significant relationship was found between informant-rated apathy and depressive symptoms although informant-rated apathy negatively correlated with executive function performance on the ToH puzzle. Furthermore, our patients underrated their apathy symptoms when compared to how informants rated their apathy. The prevalence levels for apathy obtained from patient rated scores were also below the range reported in other studies on similar patients using clinician or informant versions of the AES or different apathy instruments (e.g., [7, 8, 12]). The informant-rated prevalence rate for apathy symptoms in our study (57.5%) is similar to prevalence rates found in other studies (e.g., [29, 49–51]). 

Patient-rated apathy scores have also shown positive correlations with depressive symptoms in other studies. For example, Veranese et al. [6] found a positive but weaker correlation between apathy symptoms and depressive symptoms (Pearson index = 0.3; *P* < 0.07) when patients rated their own apathy symptoms. See also [36]. The evidence suggests that patients tend to underrate their apathy symptoms [8]. Furthermore, informant ratings of apathy symptoms are thought to provide a more reliable and valid measure of apathy than patients' self ratings [36]. Studies have also shown that of the three AES versions, the patient-rated version has the least favourable psychometric qualities [33]. 

There are suggestions that part of the apathy profile includes a dimension of diminished or lack of insight, and a tendency to minimise or deny dysfunction [21, 37, 52]. Lack of insight is a possible reason why patient ratings for apathy were lower than informant evaluations. Currently, the exact nature of the relationship between lack of insight and apathy symptoms is poorly understood. For instance, a close association between apathy symptoms, cognitive deficits, and lack of insight has been reported in numerous studies [5, 6, 53–55]. Relatedly, loss of insight into one's cognitive and functional problems (anosognosia) is prevalent in most of the disorders in which apathy is a common neuropsychiatric syndrome (see [37, 56–58]). Furthermore, apathy and anosognosia are often associated with frontal lobe dysfunction and are both sensitive to frontal lesions (e.g., [37, 59, 60]). It is important to note that 90% of the patients in our study had frontal lesions, and this could have contributed to the pattern of our results. 

It is not clear whether the validity and reliability issues raised against patient-rated apathy would apply to patient-rated depressive symptoms. The question is why, for example, anosognosia or lack of insight would affect patient self-evaluations on apathy and not on depression. It is however important to note that depressive symptoms scales used in this study have enjoyed wide validation and reliability testing and standardization and have demonstrated good psychometric properties. This is not the case with the AES-S. But more importantly, depression is largely a patient's subjective evaluation and experience of his or her situation. Conceptually, a patient's self evaluation on depression scales tapes into this subjective experience of his condition. On the other hand, in apathy, we are more interested in the objective assessment of loss of function. Apathy scales should be able to give an objective assessment of the patient's motivational state, and the functional deficits around interpersonal relationships and the initiation and maintenance of goal directed activity, whereas depression scores necessarily target the patient's subject experiences. Also, apathy is seldom distressing to the patient, but often exerts significant caregiver burden, while depression distresses the patient [61]. These factors may help explain why our patients underestimated their apathy symptoms. 

The low incidence of depression in our sample is in line with results from other studies on similar patients [28]. It is however possible that in chronic samples, depressive symptoms dominate the clinical picture in the early days after injury, and improve significantly as patients recover, while on the other hand apathy symptoms become a more common feature with time [62]. Since all our patients were at least 6 months after-injury, this could explain the low depressive symptoms incidence, and the comparatively higher prevalence of apathy. These results have important implications for clinical intervention and patient rehabilitation (see [63]). 

While one can argue that patients may have a better understanding of their own internally generated cognitive processes, we enhanced the sensitivity of the informant evaluations by administering them as a structured interview. Administering the AES-I in this manner has been shown to enhance its reliability and validity [4]. Higher informant-rated apathy scores were also associated with executive function deficits on the ToH, which further supports our view that patient-evaluated apathy may have underestimated the presence and levels of apathy. Understanding the variables that mediate the relationship between apathy and depressive symptoms will help outline the syndronomic position of apathy and inform diagnosis and intervention. In our study, the positive correlation between depression and patient-rated apathy is most likely a superficial relationship which reflects more on other patient-related variables such as lack of insight than anything else. More work is required in investigating how patient insight relates to apathy symptoms. Our study also emphasises the need for caution in interpreting patients' self-evaluations on apathy. 

## 5. Conclusion

This study demonstrates that the choice of apathy assessment tools can produce differential results on the relationship between apathy and depression. It also raises questions on the clinical utility of patient ratings when assessing apathy. These results offer a possible explanation to some of the mixed results that studies on the relationship between apathy and depression have produced. As this research demonstrates, one of the factors involved may be something to do with who rates the apathy symptoms. Future studies may also include independent measures of lack of insight in order to control for its effects on the relationship. 

## Figures and Tables

**Table 1:** Patient characteristics and cause of brain injury.

Etiology	*N*	Sex M = male F = female	Age Mean and (St. Dev)
Cerebrovascular accident	24	M = 15; F = 9	54.44 (9.73)
Head injury	14	M = 11; F = 3	48.25 (15.27)
Anoxia	5	M = 5; F = 0	49.80 (11.05)
Herpes simplex encephalitis	6	M = 5; F = 1	42.50 (8.60)

Total	49	M = 36; F = 13	50.89 (13.94)

**Table 2:** Relationships between apathy and depression scores.

		AES-S	AES-I	NPI	HADS-D	BDI
	Correlation coefficient	1.000	.266	.275	.480**	.417**
AES-S	Sig. (2-tailed)	·	.093	.085	.001	.004
	*N*	40	40	40	40	40
	Correlation coefficient		1.000	.707**	.156	.058
AES-I	Sig. (2-tailed)		·	.000	.330	.718
	*N*		40	40	40	40
	Correlation coefficient			1.000	−.037	.078
NPI	Sig. (2-tailed)			·	.819	.630
	*N*			40	40	40
	Correlation coefficient				1.000	.554**
HADS-D	Sig. (2-tailed)				·	.000
	*N*				40	40
	Correlation coefficient					1.000
BID	Sig. (2-tailed)					·
	*N*					40

**. Correlation is significant at the 0.01 level (2-tailed).
